# Relay discovery and selection for large-scale P2P streaming

**DOI:** 10.1371/journal.pone.0175360

**Published:** 2017-04-14

**Authors:** Chengwei Zhang, Angela Yunxian Wang, Xiaojun Hei

**Affiliations:** 1 School of Electronic Information and Communications, Huazhong University of Science and Technology, Wuhan, Hubei, China; 2 Akamai Technologies, inc., Cambridge, Massachusetts, United States of America; University of Rijeka, CROATIA

## Abstract

In peer-to-peer networks, application relays have been commonly used to provide various networking services. The service performance often improves significantly if a relay is selected appropriately based on its network location. In this paper, we studied the location-aware relay discovery and selection problem for large-scale P2P streaming networks. In these large-scale and dynamic overlays, it incurs significant communication and computation cost to discover a sufficiently large relay candidate set and further to select one relay with good performance. The network location can be measured directly or indirectly with the tradeoffs between timeliness, overhead and accuracy. Based on a measurement study and the associated error analysis, we demonstrate that indirect measurements, such as King and Internet Coordinate Systems (ICS), can only achieve a coarse estimation of peers’ network location and those methods based on pure indirect measurements cannot lead to a good relay selection. We also demonstrate that there exists significant error amplification of the commonly used “best-out-of-*K*” selection methodology using three RTT data sets publicly available. We propose a two-phase approach to achieve efficient relay discovery and accurate relay selection. Indirect measurements are used to narrow down a small number of high-quality relay candidates and the final relay selection is refined based on direct probing. This two-phase approach enjoys an efficient implementation using the Distributed-Hash-Table (DHT). When the DHT is constructed, the node keys carry the location information and they are generated scalably using indirect measurements, such as the ICS coordinates. The relay discovery is achieved efficiently utilizing the DHT-based search. We evaluated various aspects of this DHT-based approach, including the DHT indexing procedure, key generation under peer churn and message costs.

## 1 Introduction

Peer-to-peer networks often utilize relay nodes to provide various networking services [1–4]. These services include the traversal of Network Address Translator (NAT) [1], content search [2], application level multicasting and medium caching [3]. In particular, the end-to-end performance often improves significantly if relays are selected appropriately based on their network locations because the traditional IP routing is often not efficient from the end-users’ perspective. This performance gain by relays can be supported by the measurement results [5] that the triangle inequality may not be held for the link delays in the network distance space. Almost 47% detour paths are shorter than or equal to direct paths on the Internet [6].

An application relay is an intermediate node which performs application-layer packet forwarding between end-hosts. In a peer-to-peer network, relays can be designated peers or part of the infrastructure used by the overlay. The increasing use of application-level relays imposes potential costs to end-users and network operators. Relay nodes may also become communication bottlenecks since they introduce additional packet delay and loss. The service performance of peers may suffer significantly if relay nodes are not selected appropriately. In a large P2P overlay there may exist millions of peers and many peers can serve as relays. It is not trivial to discover a sufficiently large relay candidate set. Among many potential relays, it may also impose significant communication and computation overhead to select the optimal relay. In this paper, we are motivated to investigate the relay discovery and selection problem for large-scale P2P streaming.

A streaming session between peers often lasts for long durations. If relays can be selected appropriately with location-awareness at the beginning and be switched adaptively during sessions when necessary, the streaming service performance may be enhanced significantly. The modern P2P systems are now being equipped with various measurement features, such as the Azureus file sharing network using Internet Coordinate Systems (ICS) [7]. Third-party measurement platforms may also assist the measurement or predication of the network performance [8]. The emerging software defined measurement techniques are promising to enable adaptive and efficient implementations of passive and active network measurements which can be controlled dynamically at run-time [9]. Assume that peers’ network location can be measured directly or indirectly using these utilities. With the assistance of these measurements, the relay discovery and selection can be achieved more efficiently and accurately to meet specific service requirements of the P2P applications.

Structured overlays exhibit excellent scalability in managing millions of peers. The conventional approach for service discovery in structured overlays is to embed service description documents into keyword fragments called *strands* and index these *strands* separately into the Distributed Hash Table (DHT) [10]. Services with a high occurrence frequency in the overlay are usually not effectively indexed as *strands* of service description documents because these indices are heavily clustered at a few points in the DHT, and there are many *strands* per service description. In addition, such *strand* indexing may not be well suited to the service discovery based on location or other meta information. We explore the possibility to index the peer’s properties, such as location, work load and availability, when the DHT is constructed; then utilize the search capability offered by the DHT to discover relay candidates efficiently.

After the relay candidates are found, different selection methods may incur significantly different communication and computation overheads. In addition, the selection process may introduce errors in identifying an appropriate relay. In this paper, we aim to meet the challenges in relay discovery and selection and make the following contributions:

- The direct and indirect measurements vary significantly in terms of timeliness, overhead and accuracy. The measurement process inevitably introduces errors; these errors may impact the performance of the relay discovery and selection. Based on a measurement study, we demonstrate that indirect measurements, i.e., King and Internet Coordinate Systems (ICS), often lead to coarse estimation of peers’ network location. To the best of our knowledge, such an in-depth empirical error analysis has not been reported in the literature.
- Usually the optimal relay is selected as the *best* relay out of the *K* candidates. We demonstrate that there exists significant error amplification of this basic “best-out-of-*K*” selection methodology using three RTT data sets publicly available.
- We propose a two-phase approach to achieve efficient relay discovery and accurate relay selection. In this approach, indirect measurements are used to narrow down a small number of high quality relays and the final relay selection is refined based on direct probing. We conduct a measurement study on PlanetLab and evaluate the performance of the proposed approach.
- This two-phase relay discovery and selection scheme can be implemented using the Distributed-Hash-Table (DHT). The peers’ properties, i.e., network location and work load obtained by indirect measurements, are indexed as the node keys in the DHT. The relay candidates can be discovered efficiently by querying these keys over the DHT. We also analyze the message cost of the proposed DHT-based relay discovery and selection.

The remaining of the paper is organized as follows. In Section 2, we describe the relay discovery and selection problem in general and review some commonly used relay selection techniques using direct probing. In Section 3, we evaluate the performance of the relay discovery and selection using indirect measurements. To understand the coarse estimation of network location using indirect measurement, we conduct an error analysis in Section 4. We also analyze the error amplification effect in the “best-out-of-*K*” selection in this section. We further propose a two-phase relay discovery and selection approach in Section 5. In Section 6, we discuss how to implement this two-phase relay discovery and selection using the DHT. Then, we provide an overview of the related work in Section 7. Finally, we conclude the paper in Section 8.

## 2 Relay discovery and selection

### 2.1 Problem definition

**Fig 1:**
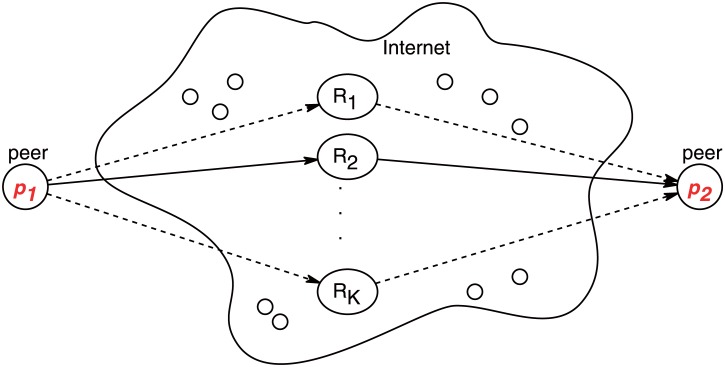
A pair of streaming peers connected via relays.

We consider a large-scale P2P network for streaming applications with *N* peers. We assume that peers and relays are also the overlay nodes of this P2P network. In such a large-scale overlay, there are many thousands, perhaps millions, of relay candidates, denoted as *M*. To serve as a relay candidate, a peer must have a public Internet address and have sufficient bandwidth capacity. We expect that the population of relay candidates to be a significant portion of the overlay population so as to achieve load balancing and sufficient capacity for many concurrent streaming sessions. A peer usually does not know the complete relay candidate set. We denote *K* as the number of the relay candidates which a peer has discovered. As shown in Fig 1, peer *p*_1_ initiates a streaming session with a remote peer *p*_2_ using relays. Peer *p*_1_ and *p*_2_ must mutually agree on selecting an intermediate peer to serve as a relay for NAT traversal or other purposes. We call these prospective peers *candidate relays*, i.e., *R*_*i*_(*i* = 1, …, *K*). The process of identifying these candidate relays is *relay discovery*. *Relay selection* is the process to choose a relay or multiple relays from the set of candidate relays to meet service requirements of P2P applications. Although multiple relays may be used along a path, we only study the case of a single relay for simplicity in this paper. In practice, a peer may engage in many sessions to different peers. It is prerequisite to maintain a large set of candidate relays in order to select a high-quality relay for a session because session endpoints may be arbitrarily distributed in an Internet-scale overlay.

The goal of the relay discovery and selection is to optimize the service performance of the P2P applications and minimize the total incurred cost. This service performance is usually application-specific, such as minimizing the delay or maximizing the TCP throughput [11]. For example, TCP throughput *B* over a link is estimated as *B* ∼ 1/(*d* · *p*^1/2^) [11], where *d* is the link delay and *p* is the packet loss probability. Assume a homogenous packet loss probability on all links. The effectiveness of the relay for increasing the TCP throughput depends on its proximity to the mid-point position on the end-to-end path between two end-points. The cost also includes the communication and computation overhead to accomplish the discovery and selection tasks. There are different approaches for a peer to discover relay candidates as follows:

- Manual configuration: The simplest way to discover relays is that each peer makes the explicit advertisement to other peers for its availability for serving as a relay in its overlay routing table; other peers search relay candidates via looking up the entries of the overlay routing tables of neighboring peers.
- Relay bootstrap: A peer in the overlay can register with a bootstrap server for relay candidacy. When a new peer joins the overlay it can receive a configured list of relay candidates from this bootstrap server.
- Relay gossip: Peers have a routing layer which typically maintains address information about other peers in the overlay, and this information is updated when peers join and leave the overlay. Peers may utilize gossip-like protocols to harvest additional relay candidates from other peers.
- DHT-based discovery: In structured P2P networks using the DHT, peers can identify themselves as a relay candidates for discovery by placing an advertisement in the DHT. The index key can be hashed based on a geographic grid or other grids. We will discuss in detail the DHT-based relay discovery based on a grid using ICS coordinates in Section 3.

Given such a list of candidate relays, the network positions of these relays relative to end-points is important to achieve a high-quality relay selection. Two basic approaches are commonly used to obtain this location information: direct probing and indirect measurement.

### 2.2 Relay selection using direct probing

Many Internet applications have adopted the Round-Trip Time (RTT) between hosts to approximate the location of hosts on the Internet. RTT can be measured directly using the utility ping or RTTometer [12]. Suppose that two peers intend to find an appropriate relay. They need to measure the RTT between each candidate relay and two end-points, *RTT*_1_ and *RTT*_2_. This requires at least two probes per relay candidate, one for each segment.

To select an optimal relay, the straight-forward idea is to probe all the relay candidates in *brute force*; then select the best relay over all the possible candidates. However, it is not feasible to maintain the global knowledge about all the relay candidates in a large-scale network. It is, nevertheless, possible for a peer to conduct RTT measurements for a number of other peers over time. Each peer may maintain a relay selection history for future sessions, and peers in the vicinity can share this historic relay list. This *relay caching* technique is helpful in minimizing the measurement overhead. Over time, peers learn the network location information of other peers via measurements. The measurement overhead can be averaged over time so that the overhead becomes affordable. On the other hand, the other endpoint must also cache RTT measurements, and the set of cached measurements must be shared among common peer pairs; by the utility of the cache in the measurement, it would decrease the overhead through the entire relay selection procedure. Otherwise, cache missing requires additional measurements; then, it reduces the utility of the cache.

Let the probability that a peer is a relay be *f*, and the size of any peer’s relay candidate cache be *m*. Suppose one peer with a relay cache size *m* intends to establish one relay path with another peer in a *N* peers overlay network. The probability that any relay candidate cached locally is also in the cache of the remote peer, is (*m*/*f* · *N*) and the expected size of the conditional intersection of these two caches is *E*(|*m*_1_ ∩ *m*_2_|) = *m*(*m*/*f* · *N*). For example, when *N* = 1, 000, 000, *f* = 0.1, *m* = 100, the expected number of relays appearing in both caches is very small, *E*(|*m*_1_ ∩ *m*_2_|) = 0.1. Therefore, caching is not an effective approach to decrease the measurement overhead for the relay selection.

*Random sampling* is another technique to minimize the cost in relay selection. It can be conducted as follows:

- *p*_1_ randomly chooses *k* peers from its local relay candidate list {*R*_1_, …, *R*_*K*_}.
- If *p*_1_ does not have recent RTT measurements for segments [*p*_1_, *R*_*k*_] and [*R*_*k*_, *p*_2_], it performs the RTT measurements. It sends at least one probe to each of the *R*_*k*_ peers. Each probe consists of request and response messages between the pair of peers. RTT measurements are conducted between [*p*_1_, *R*_*k*_] and [*R*_*k*_, *p*_2_].
- Relay *R*_*k*_ may check whether it has sufficient capacity to serve as a relay. This is the relay admission control test.
- If *R*_*k*_ has sufficient capacity and availability, then *R*_*k*_ replies to the probe and includes the RTT measurement(s) to *p*_2_.
- Select the relay *R*_*p*_ to optimize the performance function, i.e., $R_{p}=\underset{R_{k}\in \left\{R_{1},\ldots R_{K}\right\}}{argmin}\left(RTT_{p_{1},R_{k}}+RTT_{R_{k},p_{2}}\right)$.

Thus, there are at least 4 probe messages per relay, and it takes at least 2 * *RTT*_*avg*_ to complete the measurements if only one probe per segment is used.

In summary, the brute-fore direct probing does not scale for relay selection in large overlays, and neither caching measurements nor random sampling eliminates the need for a large amounts of probing.

## 3 Relay discovery and selection based on indirect measurements

Direct probing introduces significant measurement overhead for large-scale overlays. In this section, we study the relay discovery and selection based on indirect measurements. In particular, we explore the possibility using the ICS coordinates for assisting relay discovery and selection because the ICS-based approach achieves a good tradeoff in measurement overhead and computation cost.

### 3.1 Network location estimates

In a large-scale P2P network, the traffic overhead of measuring RTT between peers directly can be huge and these measurement may not be ready immediately for use. Instead, the indirect measurement of network location is a more practical approach with less measurement overhead. These indirect measurement techniques include Internet Coordinate Systems (ICS) [13–15], Autonomous System (AS) topology [16, 17], or DNS servers using the King tool [18].

In ICS, the network location of a host is mapped to a coordinate vector in a Euclidean space or other geometric spaces with a distance function. The AS information is also a good approximate of network location. An AS is usually controlled by the same Internet Service Provider. Within the same AS, two hosts are close to each other because network resources are usually over-provisioned within the AS and the congestion often occurs at the boundary of the AS. The delay between two ASes can approximate the distance between two end-hosts in different ASes. Similarly, a host is located closely to its authoritative DNS server. The delay measurement tool, King [18], estimates the delay between two end-hosts using the delay between the two DNS servers of these two hosts.

### 3.2 Performance evaluation

We evaluate the performance of relay discovery and selection using three data sets publicly available with indirect RTT measurements. These three data sets are summarized in Table 1. In the OSU data set, we obtained the IP addresses of the Gnutella peers used in [17]. After eliminating hosts in the same DNS domain, we utilize the King [18] tool to measure RTT between each pair of remaining hosts. For our experiments, we retain those hosts that have a full-rank RTT matrix between themselves.

**Table 1:** RTT data sets.

	MIT [15]	Cornell [19]	Ohio State (OSU) [17]
# endpoints	1700	2500	1103
Source	Gnutella	DMOZ and Yahoo directories	Gnutella
RTT Measurement	Median of King measurements, filter out 10% of outlier nodes	Median of 10 King measurements	Median of 6 King measurements, remove nodes with incomplete
Average RTT	180 ms	74 ms	136 ms
Measurement date	2004	May 2004	Jan 2007
Measurement Period	1 week	9 days	1 day

Using the RTT data sets in Table 1, we conduct relay experiments to use one relay for minimizing the delay between a pair of peers. We select the peer pairs with the direct RTT > 200 msec in the above three data sets. We run different relay discovery and selection algorithms to find one relay for the selected peer pairs. We use the Vivaldi algorithm [15] to compute the ICS coordinates for each host using the King-measured full-rank RTT matrix as the input. If not specified explicitly in the following experiments, in computing the Vivaldi coordinates, we set the number of random neighbors to be 32 and each peer has 6 dimensional coordinates, as recommended in [15]. In computing the distance between nodes in ICS, we map the network location of a host to a coordinate vector in a Euclidean space, and the distance is computed as the Euclidean distance.

**Fig 2:**
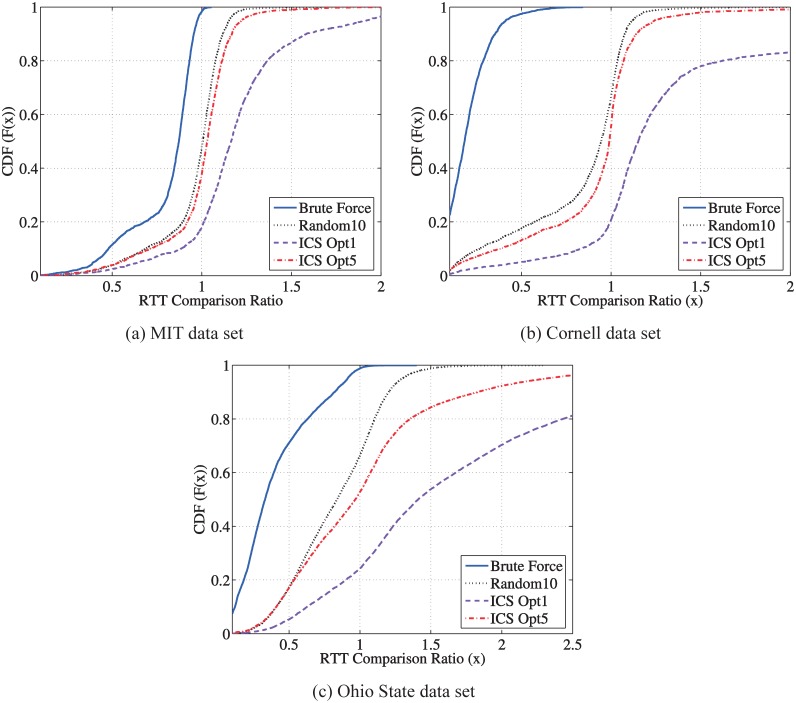
Performance comparison between different relay selection methods.

To evaluate the delay performance using a relay for one peer pair, we define RTT comparison ratio as the ratio between the total end-to-end RTT via the selected relay to the direct RTT. Fig 2 shows the Cumulative Distribution Function (CDF) of the RTT comparison ratio. Note that when this ratio equals 1, the peer pair does not achieve any benefit to decrease the RTT using a relay. The “brute force” method serves as a benchmark of the optimal relay selection in that all the relay candidates are examined and the one is selected which has the minimal end-to-end RTT for a peer pair using this relay.

Over all the three data sets, we observe that the benefit using relay in decreasing RTT is significant. For example, in the OSU data set shown in Fig 2(c), around 70% of the peer pairs can find one relay so that the end-to-end RTT can be decreased by 50%. For the “ICS Opt 1” method, each pair of hosts select the relay, via which they have the minimum end-to-end distance in the ICS space. The “ICS Opt 1” curve shows the RTT performance of this method. Though there is still delay benefit using relay, i.e., *only*23% of the peer pairs finds a relay with decreased RTT in the OSU data set, the delay performance of this ICS-based relay selection is far from the optimal. This observation motivates our in-depth study on the two-phase relay discovery and selection, which will be discussed in Section 5. To reveal some flavor of this method, we introduce “ICS Opt 5”, in which each pair of hosts select five best relays, via which they have the five smallest end-to-end distance in the ICS space; then they choose the one out of those five as the final relay, via which they have the smallest RTT value using the King measurements. The “ICS Opt 5” curve shows the performance of this two-phase method. We observe a significant performance gain over the “ICS Opt 1” selection, i.e., in the OSU data set the peer pairs with the RTT decreasing effect of the relay increases from 23% to 52%. On the other hand, the additional measurement overhead of “ICS Opt 5” increases only marginally comparing to “ICS Opt 1”.

## 4 Why does indirect relay selection perform poorly?

As shown in Section 3.2, the relay selection using indirect measurements often leads to unsatisfactory performance. One possible reason is that those ICS coordinates are computed using the indirect RTT measurements of the King tool. In this section we will examine “why does indirect relay selection perform poorly?” based on a measurement study. We find that the accurate estimation of path RTT plays an important role in selecting an optimal relay.

### 4.1 RTT measurement setup

To illustrate the importance of the measurement accuracy, we conduct RTT measurements on PlanetLab nodes with two tools, RTTometer and King. RTTometer is a direct measurement tool the same as the traditional *ping*, except that it uses the TCP handshake mechanism instead of ICMP packets so as to avoid packets filtering and rate-limiting configured by most firewalls and routers. The King tool essentially approximates the delay of two end-hosts with the delay between the respective DNS servers of those two end-hosts.

We deploy RTTometer and King in randomly selected 260 accessible PlanetLab nodes as the end-hosts and probe all other PlanetLab nodes for RTT measurements. Each end-host probes other nodes one by one. After one round finishes, a new round starts. To achieve a fair comparison on the measurement accuracy, we measure RTTs between each pair of hosts using RTTometer and King during the same period time. For each end-host, it first probes one remote node with RTTometer 10 times with the inter-probe interval of 500 msec; then we use King to estimate RTTs of this node pair for 10 times with the interval of 1 second. After one remote node is finished, a new remote node is selected for RTT measurements. The measurement interval between RTTometer measurements and King measurements is set to be less than one minute, thus the time effect on the measurement accuracy is reduced. Each pair measurement takes less than 1 minute to finish, and it takes less than 1 day to finish the measurements of all pairs. The total data sets of the RTTometer and King measurements are summarized in Table 2.

**Table 2:** Our RTT data set over PlanetLab nodes.

	RTTometer	King
# end-points	706	733
# pairs	105,911	122,337
Workload	10 measurements	10 measurements
Inter-probe Interval	500ms	1 second
Average RTT	151.72 ms	138.89 ms
Measurement Period	15 hours	

### 4.2 Inaccuracy of indirect RTT estimate

**Fig 3:**
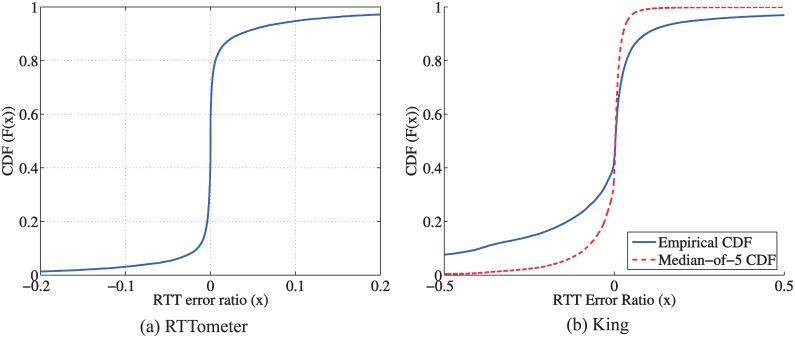
The cumulative distribution of the error ratios of the RTT measurements.

To evaluate the performance of RTTometer, we randomly choose about 8600 pairs in Table 2. For these pairs, we did not conduct any pre-process on the measurement results; instead, we take all the available results into account, 6 measurements for each. For each pair, we compute the error ratio of each RTTometer measurement with the median value,

$$\left(RTT_{*}-RTT_{median}\right)/RTT_{median},$$

where RTT_*_ is each measurement data point. In Fig 3(a) we plot the CDF of this error ratio. We observe that the error ratios closely cluster at the vicinity of zero. This indicates that the RTTometer measurement is quite accurate. The median of the RTTometer measurements can serve as a reasonably good estimate of the real RTT value of one pair. To evaluate the accuracy of the King measurements, for each pair, we take 10 measurements and compute the error ratio of each King measurement as (*RTT*_*King*_ − *RTT*_*RTTometer*_)/*RTT*_*RTTometer*_, in which *RTT*_*RTTometer*_ is the median of the RTTometer RTTs of the pair. Fig 3(b) plots this error ratio for all 74, 339 pairs with RTT between 5 msec and 2000 msec which appear in both the RTTometer and King results. As shown in Fig 3(b), the King RTTs exhibit some variation. It is an indication of the incurred measurement errors in King more severe than those in RTTometer. These errors inevitably degrade the performance of the ICS-based relay discovery and selection since the previous ICS-coordinates are computed based on the King RTTs.

### 4.3 Error amplification in best-out-of-*K* selection

The general relay selection is usually conducted in the “best-out-of-*K*” fashion in that given *K* candidate relays, the best one is selected out of these *K* candidates. We will show that the measurement errors may introduce a significant error amplification effect in this “best-out-of-*K*” relay selection. In our numerical experiments, among all the usable relay nodes, we randomly select *K* relays; the best relay is selected among these *K* relay candidates, namely, “random *K*”.

For the simplicity in the illustration, assume all relay paths have exactly the same delay as the direct path for each pair of hosts. That is, for each pair of hosts, the RTT values via different relays have the same values as the direct path. The delay measurement error follows the empirical distribution of the King measurement error as shown in Fig 3(b). Let *X*_*i*_ be the measured delay on relay path *i*, *X*_0_ be the real delay. The CDF of (*X*_*i*_ − *X*_0_)/*X*_0_ is plotted in Fig 3(b). When we conduct the “random *K*” best relay selection, the selected shortest relay path is

$$X_{K}^{*}=\min_{1\leq i\leq K}X_{i}.$$

**Fig 4:**
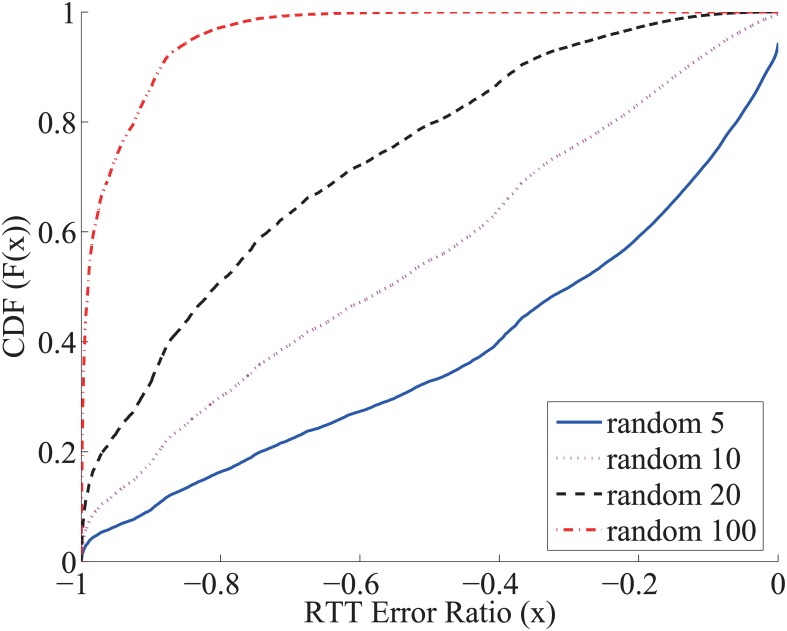
Best-out-of-*K* amplifies RTT underestimate.

The CDF of $\left(X_{K}^{*}-X_{0}\right)/X_{0}$ is plotted in Fig 4. For example, the “random 10” relay selection mistakenly reports a shortest relay path 60% shorter than the direct path 50% of the time, even though no real shorter relay path even exists. The RTT underestimates will be amplified by the “best-out-of-*K*” relay selection algorithm, in that a larger *K* mistakenly leads to a larger reported improvement.

**Fig 5:**
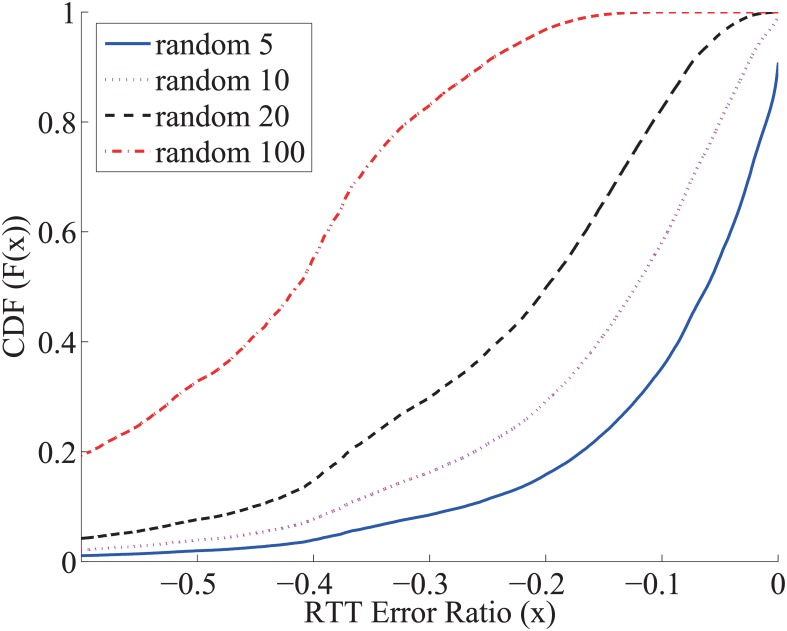
Error reduction through median-of-5.

To reduce the RTT measurement error, for each relay path, one can conduct multiple measurements and use the median value. The median-of-5 CDF curve in Fig 3(b) plots an improved RTT measurement error CDF for the median-of-5. Compared with the empirical CDF curve in Fig 3(b), the RTT measurement error has been reduced. Consequently, the error in the “best-out-of-*K*” relay selection is also reduced as shown in Fig 5. For example, the “random 20” relay selection mistakenly reports a shortest relay path 20% shorter than the direct path only 50% of the time.

Comparing Figs 4 and 5, we observe that measurement inaccuracy may introduce a significant error amplification in the “best-out-of-*K*” relay selection. If there are significant errors in the measurements, the reported “best-out-of-*K*” relay would be actually far from the real optimal value due to this error amplification. Therefore, the measurement practitioners should minimize this error so that the reported “best-out-of-*K*” relay is actually near the real optimal value.

### 4.4 Examination of the Euclidean triangular inequality assumed by ICS

As we mentioned in the previous section, ICS can be adopted for relay discovery. However, the optimal relay, along which the relay path has a shorter distance than the direct path, essentially violates the Euclidean triangular inequality which is assumed by ICS. Given a graph *G* with a set of vertices *V*, a cost function *C* is said to satisfy the triangle inequality if for all vertices *a*, *b*, *c* in the graph, *C*(*a*, *c*) ≤ *C*(*a*, *b*) + *C*(*b*, *c*). In ICS, the coordinates are calculated based on this rule. For the relay selection to minimize the delay between peers, we actually attempt to find a midpoint node that can reduce end-to-end delay. This is a triangle inequality violation. Nevertheless, we can still use it for network location estimation. In the relay selection problem, ICS should only serve as a guideline to locate good relay candidates.

### 4.5 Measurement-based error evaluation of indirect measurements

We have discussed the impact of the “best-out-of-*K*” approach on the accuracy of the relay selection; however, this discussion only provides some qualitative insights on this error issue. To consider the heterogeneity of the Internet paths, using our RTT measurements over the PlanetLab nodes we continue with an in-depth investigation on the errors in two popular indirect RTT measurements: King and ICS. We apply the Vivaldi algorithm [15] with the number of random neighbors of 32 to compute 6-dimension ICS coordinates for each host. The RTT matrix used in the coordinate computation is measured by RTTometer instead of King since the errors incurred by King may have negative impacts on the performance of the ICS-based relay selection. In addition, the RTT matrix consists of 494 selected nodes. Each of these nodes have at least 100 neighbors; hence, the Vivaldi computation error can be reduced. Finally, we obtain a 494-by-494 ICS Euclidean distance matrix.

#### 4.5.1 Absolute errors

Among the measured Internet paths in Table 2, we selected 15, 761 pairs with the RTT between 5 msec and 2000 msec which appear in all RTTometer, King and ICS results. About 95% paths have RTT less than 300 msec. For each path, multiple measurements are conducted and only the median RTT value is chosen for each path.

**Fig 6:**
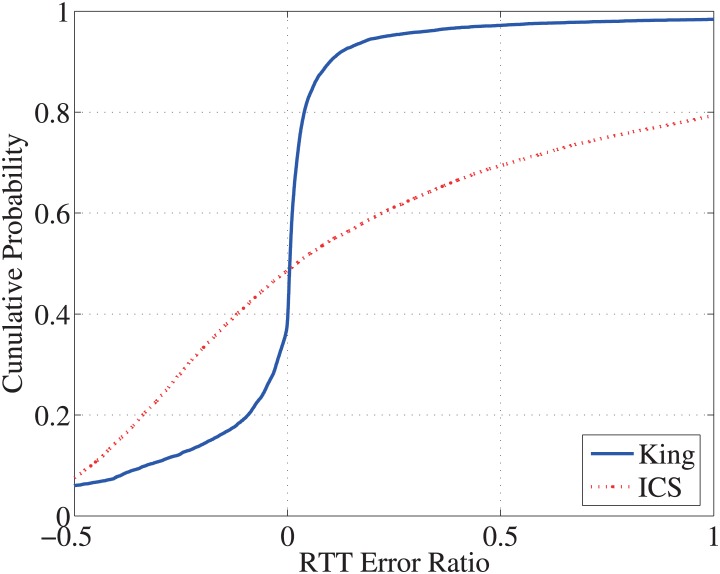
CDF of the RTT comparison ratio between indirect RTTs and the direct RTTs.

For each path, we compute the RTT error ratio as (RTT_*_ − *RTT*_*RTTometer*_)/*RTT*_*RTTometer*_, where RTT_*_ is either the King or ICS measurements. In Fig 6, we plot the CDFs of the RTT error ratios for the selected 15, 761 paths. We can observe that the ICS measurements deviate quite far from the direct RTT measurements, and this deviation is relative small for King. In Fig 6, about 90% of the King RTT measurements are within 50% error compared with RTTometer RTTs, and about 80% results are within 20% error. However, for ICS, only a small percentage (<20%) of all paths are near the RTTometer RTTs, where about 20% paths are within 20% error, and about 60% paths are within 50% error.

**Fig 7:**
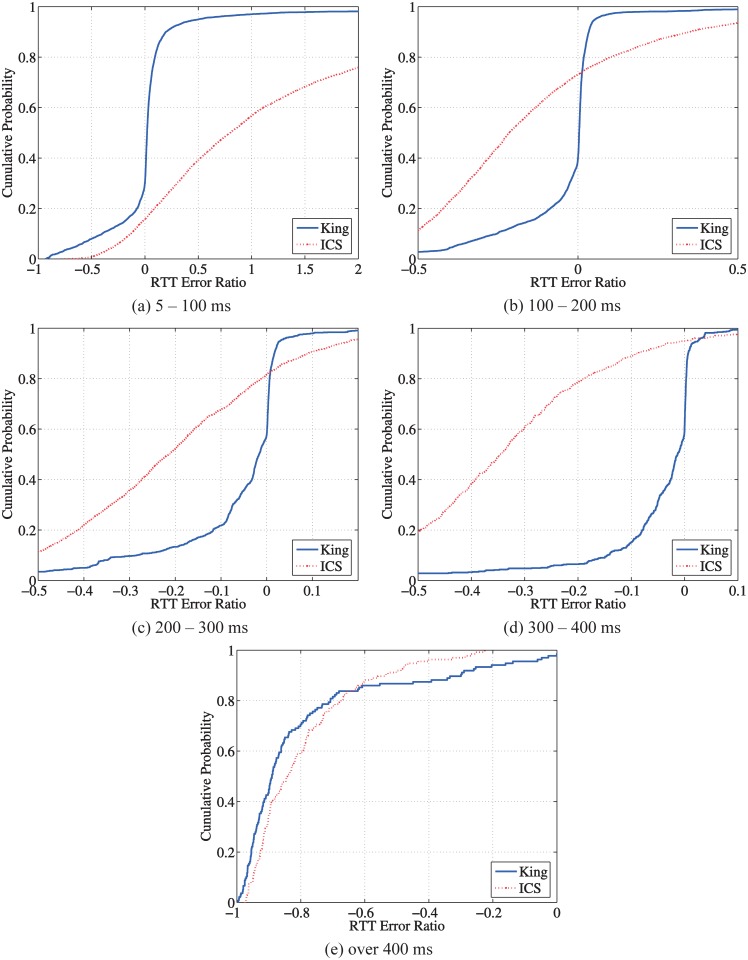
Absolute errors of indirect RTT estimation.

To exam the absolute errors in King and ICS, we plot RTT error ratios in finer RTT granularities. In Fig 7, we plot the CDFs of the RTT error ratios of ICS/King measurements in different RTT ranges. Among all 15, 761 paths, there are 7, 259 paths with the RTT in [5, 100) msec, 6, 251 paths in [100, 200) msec, 1, 453 paths in [200, 300) msec, 662 paths in [300, 400) msec, and 136 paths over 400 msec. As a rule of thumb, these indirect measurements perform better for those paths with the RTTs in [100, 400) msec. In addition, King performs more accurately than ICS. For example, for those pairs in [200, 300] msec, King has about 90% paths with error less than 20%, while ICS has only about 45%. In particular, both King and ICS underestimate those paths with larger RTTs (> 400 msec).

To investigate the causes of the King errors, we randomly selected several node pairs, and measured the RTTs using RTTometer and King with a closer examination. We review the measurement details, i.e., the RTT between the end host and its DNS server, whether the corresponding DNS server is in the vicinity of an end host. We identify two most important causes to the reported errors in the King measurements:

- RTTometer: The RTTometer measurement client probes remote nodes from source nodes directly. The measurement results may be impacted by the congestion level of the end-hosts. During the measurements, we find that when the CPU of a PlanetLab node is highly occupied, i.e., 80% or higher, the measured RTTs using RTTometer is unreasonably large. This may be due to random packet sending or receiving delay at the kernel of the operation system. However, the King measurements are not effected by the operation of end-hosts. The King measurements usually underestimate those paths with larger RTTs. In addition, packet loss, socket errors, or destination unreachable errors sometimes occur in RTTometer measurements. As a result, we exclude some outlying results before analysis to minimize the effects of those measurement errors.
- King: During the King experiments, we find that there are several cases that potentially introduce errors in the King measurements. First, when one of the host pair or both are located far from its authoritative name server, the measured RTTs using King usually have significant difference with the RTTometer results. This observation is consistent with the results reported in [18]. Second, some hosts may have multiple authoritative name servers in different network zones. When recursive queries used in King are sent to these hosts, the King measurements may have a large variation since a different DNS server may be used to respond to each DNS query.

#### 4.5.2 How predicative indirect measurement based selection is?

In summary, we demonstrate that there exists inherent inaccuracy in indirect RTT measurements. As we analyze in section 4.3, the measurement inaccuracy will be amplified by the “best-out-of-*K*” relay selection. The previous experiment results show there are always unavoidable errors for indirect measurement. Due to these two reasons, the relay discovery and selection using pure indirect measurements is unlikely to achieve very good performance although it incurs small measurement overhead. Nevertheless, the indirect measurements still provides a coarse characterization of peer locations. They provide good guidelines in locating relay candidates. As we demonstrate in Section 5, we propose a two-phase relay discovery and selection approach to achieve a good tradeoff between measurement overhead and selection accuracy.

## 5 Two-phase relay discovery and selection

### 5.1 Methodology

The proposed procedure consists of two phases: relay discovery and selection refinement. In the relay discovery phase, we utilize the indirect measurements to identify a small set of high-quality relay candidates with small measurement overhead. Indirect measurements are utilized to narrow down the set of *K* relay candidates to *k*′ < *K*. In the phase of relay selection refinement, the end-hosts directly probe each *k*′ relay candidates for RTT measurements; then select *r* relays out of *k*′ candidates which pass an admission test. Among the *r* relays, the one, which leads to the best application performance, is selected.

### 5.2 Performance improvement

In our experiments, the goal is to find a relay for minimizing the delay between a pair of peers. We consider two types of indirect RTT measurements: King and ICS. The RTT measurement is conducted using PlanetLab as summarized in Table 2. In the ICS-guided relay discovery, the ICS coordinates of the peers are computed using the Vivaldi algorithm. Instead of a single relay which has the shortest end-to-end Euclidean distance via this relay, namely, “ICS Opt 1”, a subset of relay candidates are identified so that these relays have the *k*′ shortest Euclidean distances among all the relay candidates. Then two peers of a session directly probe these *k*′ relay candidates for direct RTT measurements using RTTometer. Finally, among these *k*′ candidates, the relay is selected which has the minimum end-to-end RTT via that relay, we call it “ICS Opt *k*′”. Similarly, for the King-guided relay discovery, instead of a single relay which has the shortest King RTTs via the relay, namely, “King Opt 1”, we select a subset of relay candidates which has the *k*′ shortest King RTTs; then the final relay is selected with the minimum end-to-end RTT via this node among these *k*′ relays, namely, “King Opt *k*′”. In the following experiments, we set *k*′ = 5. For a comparison, the optimal relay is selected by directly probing all relay candidates using RTTometer in “brute force”.

**Fig 8:**
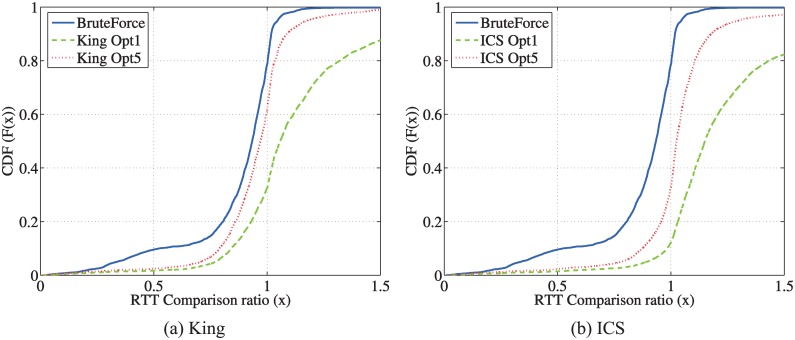
Cumulative distribution of the RTT comparison ratio using indirect measurements.

We randomly select 8, 088 paths in Table 2, which have the direct RTTs > 100 msec. For one path, two end-points select one relay to decrease the end-to-end RTT from the remaining hosts in the data set. Recall that the RTT comparison ratio is computed as the ratio between the total end-to-end RTT via the selected relay and the direct RTT. In Fig 8, we show the performance in decreasing the end-to-end delay via the relay for the two-phase selection using the indirect measurement: King and ICS. To make the results more comparable, we select the best relay out of the same relay set for all three algorithms. In Fig 8, the brute-force curve confirms the benefit of relay in that about 80% paths can find a relay path that is shorter than the direct path. “King Opt 5” achieves the a RTT reduction approaching to that of the brute-force method with much smaller overhead.

**Fig 9:**
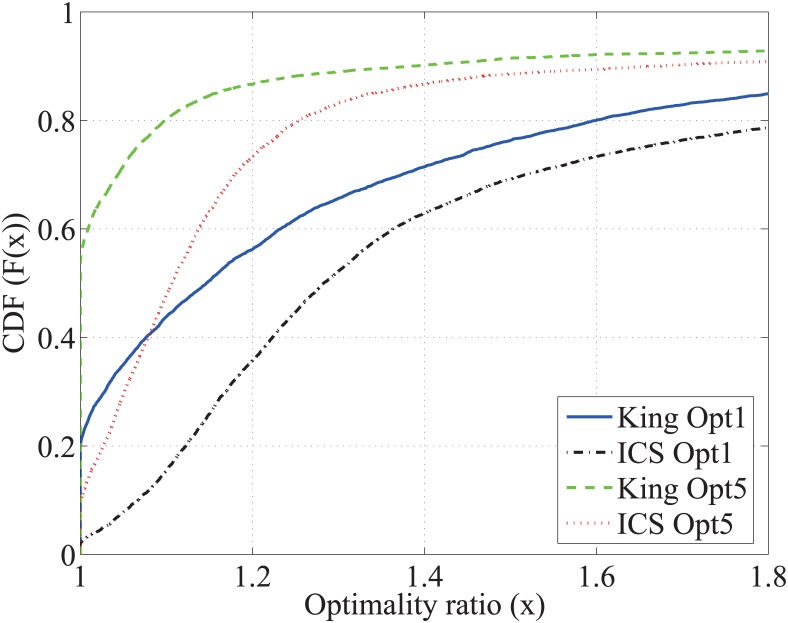
CDF of the optimality ratio using indirect measurements.

The proposed two-phase relay selection approach is essentially a heuristic approach. In the following, we evaluate the optimality of the two-phase selection using the metric of the optimality ratio, which is defined as the ratio of the end-to-end RTT using the selected relay and the RTT using the optimal relay. The optimal relay is identified using brute force. In Fig 9, we plot the CDFs of the optimality ratio of the different relay selection methods. Around 90% of the pairs can find a relay path with at most 50% larger RTT than the optimal relay path using the two-phase methods. In addition, we observe that, the King-guided relay selection has a much better performance than ICS since the King estimations are much more accurate than ICS as we showed in previous sections. This two-phase selection has significant improvements for both the King-guided and ICS-guided algorithms. In particular, “King Opt 1” can find the optimal relay path for about 20% pairs; when using “King Opt 5”, this value increases almost to 60%, which has a 40% improvement. Nevertheless, the improvement of “Opt 5” over “Opt 1” is only about 10% for ICS.

**Fig 10:**
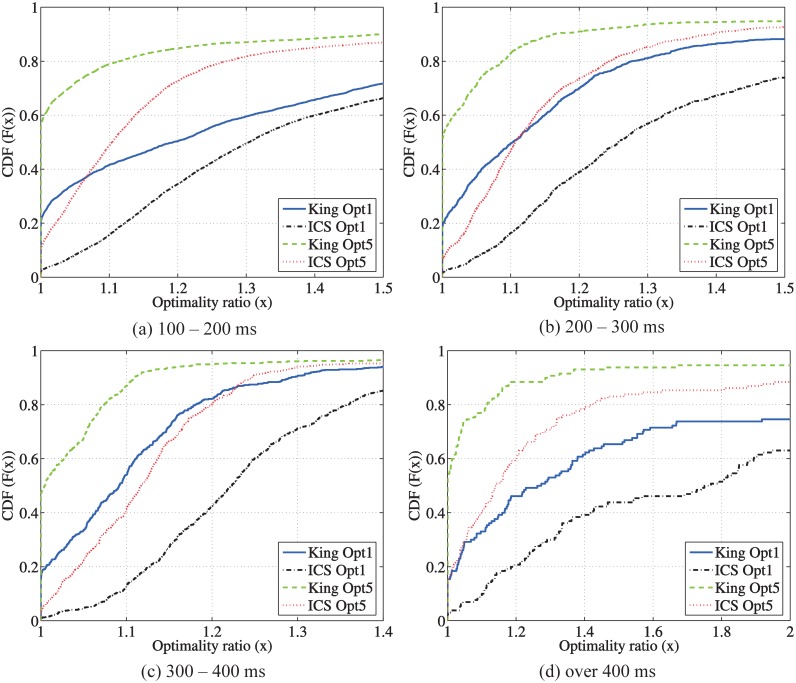
Optimality of the two-phase relay discovery and selection.

In Fig 10, we plot the CDFs of the optimality ratio for peer pairs with the different RTT ranges. Among all the 8, 088 pairs, there are 5, 933 pairs with direct RTTs in [100, 200) msec, 1, 387 pairs in [200, 300) msec, 638 pairs in [300, 400) msec, and 130 pairs over 400 msec. Both King and ICS achieve the similar performance for different RTT ranges. As shown in Fig 9, the King-guided selection achieves a better performance than the ICS-guided selection while the two-phase method has a significant improvement over the relay selection using pure indirect measurements.

### 5.3 Discussions

In this subsection, we discuss three issues in our measurement study.

#### 5.3.1 Residential access

In our measurement, we use the PlanetLab nodes. These nodes are mostly located on campus networks with the high bandwidth Ethernet access. There exist a large portion of residential broadband users in the current P2P networks. The King and RTTometer RTT measurements may incur different measurement errors in campus networks and residential networks. For example, in a campus network, the local host is likely to be close to its DNS server, while in a residential network, this distance can be far away. In addition, applications running on residential access may experience significant delay due to the access link [20]. The King tool is not able to capture this residential delay. Thus, the indirect RTT measurement using King, may introduce significant errors for residential networks.

#### 5.3.2 Impact of network dynamics

The accuracy of the RTT measurements is critical for an appropriate relay selection. The network dynamics may impact the measurement accuracy. For example, network conditions may change over time. One RTT measurement at a particular time may not represent the RTT at another time. Repeated measurements can help in minimizing this impact with additional measurement overheads.

#### 5.3.3 Implementation issues

In the two-phase relay discovery and selection procedure, indirect measurement data, such as King and ICS, should be available for narrowing down the coarse relay candidate set in the first phase. For example, for the ICS-guided selection, we need to measure the RTTs between peers in advance to compute the coordinates. If we conduct realtime RTT measurements between peers, this task becomes non-trivial when the scale of the overlay increases. We propose the following method to reduce the measurement overhead for implementation. We establish a master measurement server, which has a large collection of DNS severs and measures RTTs between each pair of DNS servers. Sharing the same spirit of the King tool, we approximate RTTs between pairs of DNS servers as the RTTs between hosts within the above two DNS domains. For each peer in the overlay, we identify the DNS server which has the longest-matching prefix with this peer. Then, using the RTT matrix between DNS servers, we can obtain the corresponding RTT matrix between peers. When a new peer joins the overlay, we extract necessary RTT information from the above RTT matrix between DNS servers without additional measurements. In this method, all measurements are conducted on the server side, and the only incurred overhead is the maintenance cost of the RTTs between DNS servers. We may update the DNS server set and re-generate the RTT matrix between the DNS servers via measurements periodically. Nevertheless, since the DNS severs do not change very often, the update period can be set sufficiently long without much accuracy loss.

## 6 DHT-based relay discovery and selection

In this section, we present the procedures to implement the two-phase relay discovery and selection efficiently using the DHT.

### 6.1 Framework overview

In a structured overlay, peers are organized in the DHT. So far, application relays do not have rich APIs for the management of relay services. Each relay candidate composes a relay service description in the DHT for advertisement as relay candidates for discovery by other peers. An advantage of relay discovery using the DHT is that these advertisements are refreshed automatically by the relay peers when relay states have changed. The characteristics of relay candidates, such as network location, bandwidth capacity and service availability, should be embedded in these advertisements for assisting appropriate relay discovery and selection. These service advertisements are managed efficiently using hash-based indexing. For example, we can construct a key as “media-relay:coordinates” for each relay candidate using ICS. Suppose that a peer pair need to identify an appropriate relay to improve TCP throughput. If the coordinates of the session end-points and the relay candidates are known, two end-points should select one of those relay candidates close to the midpoint in the ICS space between these two end-points. Note that the ICS provide a globally consistent coordinate for the overlay; however, the systems, such as Meridian [19], which rely on relative positioning, can not be used for the DHT indexing.

The framework of the DHT-based relay discovery and selection consists of the following major components. 1) Each peer estimates its network location using indirect measurement. 2) The network location information, i.e., ICS coordinates, is hashed as the keys of the relay advertisement and the advertisement is stored in the DHT. 3) When a relay is needed by a peer, this peer queries the relay advertisements in the DHT by specifying particular application requirements; then a relay candidate set is returned by the DHT routing layer. 4) The peer directly probes these relay candidates. Based on the probing results, the peer selects the best relay among them.

### 6.2 DHT indexing of ICS coordinates

In a DHT overlay, each peer probes a small number of overlay nodes and obtains its network location represented by its ICS coordinates. Relay service advertisements are indexed using these ICS coordinates. This ICS technique is also useful for discovering other communication services such as media transcoders or mixers.

We illustrate the DHT indexing procedure using an example in which a relay is used to improve the TCP throughput between two peers. These two peers have the coordinate vector *C*_1_ and *C*_2_. Relay candidates are sought in the vicinity of *C* which is the middle way between *C*_1_ and *C*_2_. We can align all coordinates on a grid and hash each relay’s nearest grid position. Then the relay discovery is achieved using the nearest grid position to *C* as the key. Each relay candidate determines its position by its coordinates, aligns the position to the nearest grid point, and then creates the key “media-relay:grid-coordinates” as its advertisement and inserts its relay service description at the position in the DHT indicated by this key. This key can be composed differently using different ICS algorithms. For example, the Vivaldi algorithm usually computes 6 dimensional coordinates; therefore, a key may be written as “media-relay:*g*_1_: *g*_2_: *g*_3_: *g*_4_: *g*_5_: *g*_6_” where each *g*_*i*_ is an integer in one of the coordinate dimensions.

### 6.3 Key distribution and query load in the DHT

**Fig 11:**
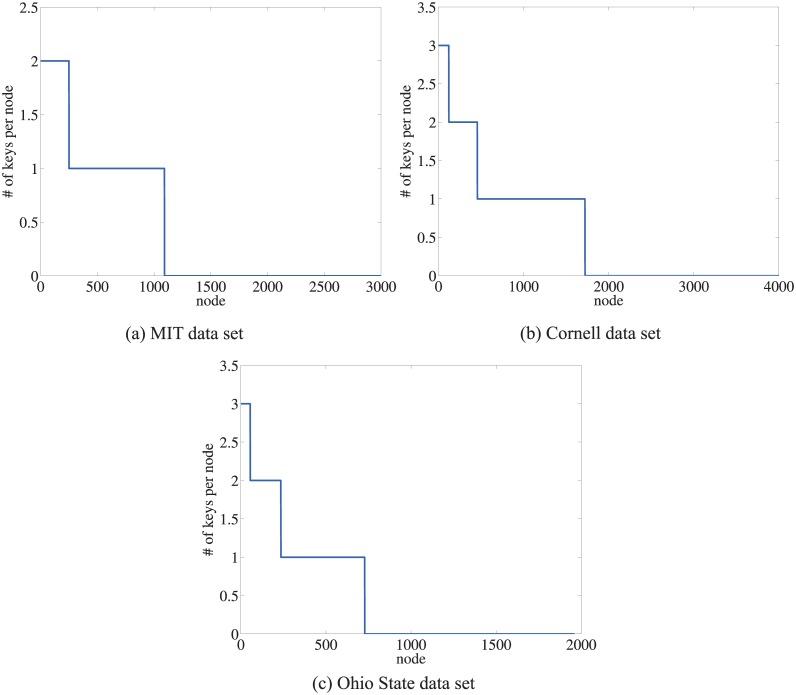
Key distributions of ICS coordinates among peers of the three data sets.

To achieve the load balancing, it is desirable that the indexing keys of the ICS coordinates are uniformly distributed in the DHT. We apply the SHA hashing function for the peers in the OSU, Cornell, and MIT data sets. This SHA hashing algorithm has also been used by many DHTs. The distribution of *N* relay keys in a DHT of 2*N* peers of the OSU, Cornell, and MIT data sets is shown in Fig 11. The average number of keys per node is about 0.5 and the maximum keys per node is 3. The keys show a somewhat uniform distribution in the DHT.

The distribution of the DHT index operations due to relay advertisement and discovery should be also evenly distributed. The distribution of the key lookup requests also depends on the distribution of end-points in the set of sessions as well as the network topology. For example, in analyzing the distribution of RTT measurements versus geographic locations, clusters are found to be associated with the US east coast, US west coast, and Europe/Asia [21]. This suggests that relay discovery load is likely to be concentrated at points within each of these clusters as well as between these clusters.

### 6.4 Churn and coordinate recalculation

A Vivaldi node periodically re-computes its coordinates at peers’ arrival and departure in the overlay. At each calculation interval, called an *iteration*, each peer updates its coordinates using the RTT values between itself and a set of other peers. Hence, the update interval depends on the churn rate of the overlay. From one interval to the next, the neighbor set of one peer in computing the Vivaldi coordinates may overlap or be entirely different. It is also desirable that these coordinate keys should be stable to avoid update cost of the keys. If the coordinates change very often, the service indices are less reliable and the index maintenance may result in significant message overhead. We evaluated the stability of the Vivaldi coordinates using three data sets tabulated in Table 1.

**Fig 12:**
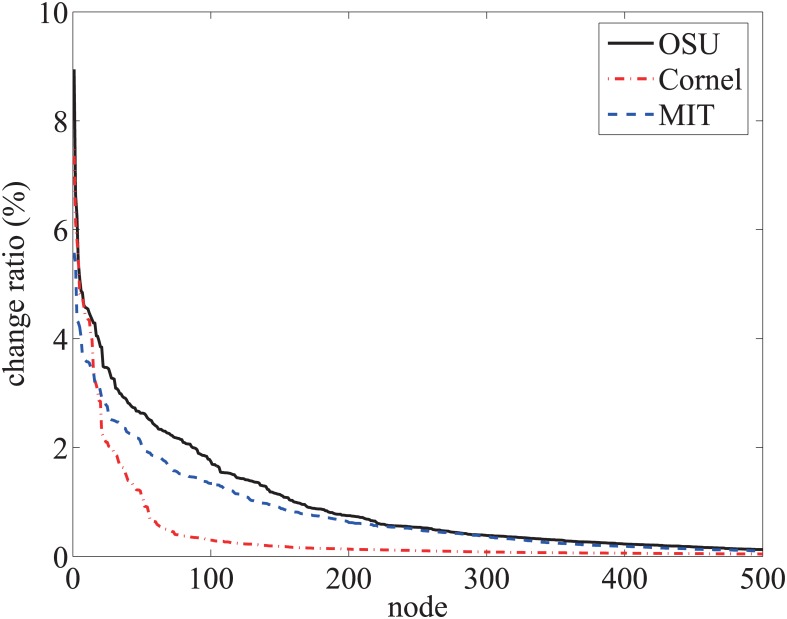
Change ratio of peers’ coordinates, starting from stable values for 90% of the peers in each data set and then merging in the remaining 10%.

We randomly select 90% of the nodes and compute the Vivaldi coordinates of these nodes. The coordinates stabilize after 15 or more iterations. After the coordinates stabilize, we then include the remaining 10% of the nodes and recompute all peer coordinates in one interval. We calculate the ratio of the distance between the coordinates for each point versus the size of the coordinate space. For all three data sets, the average change ratio of the distance was < 1%. Fig 12 plots the change ratio sorted in decreasing size for each neighbor set of the peers. The change ratio of the neighbor set, whose coordinates change exceeded the grid spacing, is 2% for the Cornell data set, 10% for the MIT data set and 16% for the OSU data set. The change rate of the relay advertisements also depends on the churn rate of relays. Relays should be substantially more stable than non-relay nodes to achieve a good performance.

### 6.5 Relay admission control

The relay peer may deploy an admission control test in that a new session is accepted only when this relay still has sufficient bandwidth capacity. The initiating peer first contacts one relay candidate to initiate the session. If the relay does not have sufficient capacity, the request is rejected and the peer has to choose another relay candidate. To minimize the probability of failed admission trials in the session setup, relays may include their current available capacity in its relay advertisement. This information is updated when new sessions are established at the relay. It can be stored either at the DHT index points as a part of the relay service description or at the relay peer itself, depending on the changing rate (*r*_*u*_) of this information versus the rate *r*_*r*_ at which the advertisement is requested. If *r*_*u*_ > *r*_*r*_, then the relay peer can store the relay service description locally and index a Universal Resource Index (URI) in its service description; otherwise, it can store the relay service description at the DHT index.

### 6.6 Message cost

In this subsection, we analyze the message cost of the DHT-based relay discovery and selection with the comparison to two other methods: direct probing without caching and direct probing with caching. We study the message overhead of the relay discovery and the relay selection, respectively. We assume that each relay is also a peer that may participate as an end-point in other streaming sessions. In Table 3, we provide the parameter notations for computing the message cost.

**Table 3:** Parameters for message cost analysis.

Symbol	Description	Example Value
*N*	number of overlay nodes	1,000,000
*M*	number of relays in overlay	500,000
*K*	number of relay candidates known to each peer	1000
*h*	number of overlay hops for *put()* or *get()* lookup	[1, 20]
*S*	number of relayed sessions initiated by each peer during the average time period	10
*k*	number of relay candidates per session using DHT-based method	*k* = 5
*j*	number of relay candidates per session using direct probing	*j* = 2*k*

When we consider the structured overlay, the DHT-based relay discovery and selection serves as a value-added service on top of the DHT overlay. Hence, we exclude the DHT construction and maintenance overhead in computing the message overhead of the DHT-based relay discovery and selection. When a peer issues a DHT lookup query for a location-based key, this lookup operation retrieves multiple relay candidates because these relays share the same grid position. We consider various DHTs with different bases, i.e., Chord (multi-hop with base 2), Pastry (multi-hop with base 2), EpiChord or OneHop (single hop). These DHTs incur different key lookup costs. We use 32 RTT probes to compute the Vivaldi coordinates which are assumed to remain stable over the time to complete *S* sessions.

One streaming session is initiated by two peers. In the DHT-based relay discovery, the message cost consists of 3 parts. 1) each peer of the total *N* peers computes its Vivaldi coordinates using the RTTs with its 32 neighbors; hence, the message cost is 32 * 2 * *N*. 2) the advertisements of the total *M* relays are stored in the DHT. Each key *put()* operation leads to *h* DHT lookups. These relay advertisements generate *M* * *h* messages. 3) A peer queries the advertisements to discover relay candidates for *S* streaming sessions. Each advertisement query leads to the key *get()* operation of (*h* + 1) DHT lookups. The total advertisement queries cost (*h* + 1) * *S* * *N* messages. After a pair of peers discover the relay candidate set, they directly probe these relays for RTT measurements and select the best relay among these relay candidates. These direct measurements result in 4 * *k* * *S* * *N* probing messages. On the other hand, for an application-specific relay selection, the relay candidates do not carry all the message workload. Its total message cost is a subset of the cost sum of the relay discovery and the per-session relay selection. In total, each of the *M* relays computes its Vivaldi coordinates using the RTTs to its 32 neighbors. Hence, the message cost for the coordinate computation is 32 * 2 * *M*. The relays also carries the direct probing cost 4 * *k* * *S* * *N* for *k* relay candidates and *S* sessions for *N* peers. Each relay needs to store its advertisement using its network coordinates as the index values in the DHT; therefore, *M* relays need *h* * *M* messages. Nevertheless, one relay only carries the message workload for the first hop; the message workload of the remaining hops are carried by other peers in the overlay. In addition, the relays do not carry the message cost due to the advertisement retrieval for discovering relay candidates. In summary, the total message workload carried by relays is computed as 32 * 2 * *M* + *M* + 4 * *k* * *S* * *N*.

When we compute the message cost for direct probing with RTT caching, we assume that the caches exchange 2 messages per relay session. In the best case, the cache intersection always produces half the relay candidates for selection and the remaining candidates require additional probing operations. For the direct probing without caching, each peer queries some bootstrap server to obtain an initial list of relay candidates. More relay candidates may be found via relay gossiping. The minimum number of messages in this case is 2 messages for each peer to discover relay candidates. Then, one peer randomly selects *j* relays from candidate list and explicitly probes these relays for *RTT*_1_ and *RTT*_2_ values; it selects the best one, i.e., $R_{p}=\underset{R_{k}\in \left\{R_{1},\ldots R_{j}\right\}}{argmin}\left(RTT_{p_{1},R_{k}}+RTT_{R_{k},p_{2}}\right)$. The probing operations lead to 4 messages for each relay candidate to obtain *RTT*_1_ and *RTT*_2_, and the total 4 * *j* RTT probe messages per session.

For direct probing with caching, the end-point peers cache RTT measurements for all known relay candidates. When one peer initiates a session, it first obtains the list of the remote peer’s relay candidates; then it intersects this list with its own relay list. If the intersection provides sufficient candidates, two end-point peers select the best one from this intersection; otherwise, each peer has to probe those missing *RTT*_2_ values, then selects the best one. To discover the relay candidates, each peer first obtains the initial candidate list. This operation generates 2 messages; then the peer probes those known candidates in the list for caching, which lends to 2 * *K* messages in total. To select the best candidate, one peer queries the cache of the remote peer using 2 messages per session. Ideally, the cache intersection returns with *j*/2 candidates. Additional probing is required for *j*/2 candidates. Thus the total message cost is 2 * *N*+2 * *K* * *N*+2 * *S* * *N*+4 * *j*/2 * *S* * *N*.

In Table 4, we tabulate a summary of the message cost of the three methods. The cost sum of relay discovery and relay selection in Table 4 represents the total message signaling overhead for all nodes during the averaging period in which all peers participate in *S* session. We also show the message workload carried by relay peers for each method in the right column of Table 4 so that the load on the relays can be compared with the different methods.

**Table 4:** Message cost of different relay discovery and selection methods.

	relay discovery	relay selection	message workload by relays
RTT probe with no caching	2 * *N*	4 * *j* * *S* * *N*	4 * *j* * *S* * *N*
RTT probe with caching	2 * *N* + 2 * *N* * *K*	(2 + 2 * *j*) * *S* * *N*	2 * *N* * (1 + *K*) + 2 * (1 + *j*) * *S* * *N*
DHT-based discovery and selection	32 * 2 * *N* + *M* * *h* + (*h* + 1) * *S* * *N*	4 * *k* * *S* * *N*	32 * 2 * *M* + 4 * *k* * *N* * *S* + *M*

In Table 5, we plug in those numerical values in Table 3 to the formula in Table 4 and calculate representative values for total message signaling and relay-specific overhead. The total message cost is the sum of the candidate collection and candidate selection message counts. It shows that using a DHT is better when multi-hop base 16 (such as Pastry) or a one-hop overlay is used.

**Table 5:** Message signaling overhead of different discovery and selection schemes for overlays with 10^6^ nodes.

	*N*	*f*	*M*	*K*	*S*	*h*	*j*, *k*	relay discovery	relay selection	message workload by relays
RTT probe with no caching	10^6^	0.5	0.5*10^6^	-	10	-	10	2*10^6^	4*10^8^	4*10^8^
RTT probe with caching	10^6^	0.5	0.5*10^6^	10^3^	10	-	10	2.002*10^9^	2.2*10^8^	2.222*10^9^
DHT-based relay discovery and selection										
Multi-hop base 2	10^6^	0.5	0.5*10^6^	-	10	20	5	2.83*10^8^	2*10^8^	2.32*10^8^
Multi-hop base 16	10^6^	0.5	0.5*10^6^	-	10	5	5	1.26*10^8^	2*10^8^	2.32*10^8^
One-hop	10^6^	0.5	0.5*10^6^	-	10	1	5	8.4*10^7^	2*10^8^	2.32*10^8^

Compared with each other, the relative performance gain of three methods remains the same when the number of the overlay nodes increases by 10 times. This can be seen from the formulas in Table 4 where the cost of each method almost linearly increases as the number of overlay nodes increases. However, if the number of session *S* is increased by 10 times, the performance of cache versus non-cache is still comparable, and as *S* increases further, the caching method has lower message overhead. Note that the overhead model optimistically assumes that each pair of caches have half intersection. The simple computation conducted in Section 2.2 shows that the half intersection in the relay caches of the two peers actually occurs unlikely. If the percentage of relays in the overlay drops from 50% to 10%, the relative performance of cache and non-cache remains similar. With the number of candidates *k*, *j* increasing, the overhead saving of DHT-based discovery becomes larger. Note that in Table 5, we set *j* = 2 * *k* since the application performance of the DHT-based discovery with 2 * *K* relays is already better than the performance of direct probing with *K* relays. If the accuracy of the ICS can be improved further, the performance gap of DHT-based discovery over direct probing will be even larger.

Assume that some percentage *q* of relay candidates are overloaded and cannot accept additional sessions. In the direct probing methods this leads to up to *q* percent additional probing messages. In the DHT-based method, each relay sends 2 * *S* status messages to update its advertisement in the DHT. As *q* increases, the message cost of the direct probing increases at a faster rate than that of the DHT-based discovery.

The DHT-based discovery has a smaller measurement overhead than the direct probing. It also has a smaller delay in relay selection. The minimum delay to complete the relay selection is 2 RTTs for direct probing, if each relay candidate can be probed in parallel. If the number of relay candidates is large, the probes need to be serialized to avoid potential interference. As a result, the delay of direct probing may increase significantly. However, the delay to complete relay selection for the DHT-based discovery is 2 RTTs for the probing plus the DHT lookup time. In the best case, the DHT lookup delay is only about 1 RTT when a one-hop overlay is used.

In addition, as we discussed previously, using the DHT-based discovery we can consider some dynamic attributes such as peer capacity and availability which are not typically considered in existing service discovery mechanisms. It makes the DHT-based mechanism more appealing.

## 7 Related work

Service discovery and selection has been conventionally studied separately in the literature, though these two problems are closely related. Pietzuch et al. evaluated a generic framework for DHT-based stream service discovery and selection in [22] so as to achieve the optimal performance jointly. We studied a special stream service, namely, the relay service for large-scale P2P streaming, as a case study in depth and provided an analysis of the message cost of the DHT-based relay discovery. The emerging overlay networks offer various communication services [23]. These services are usually discovered via querying some central server. Researchers have not pay sufficient attentions to various service discovery issues. On the other hand, the service selection problem has been investigated heavily in various contexts.

Service selection, such as relay selection, can be formulated into a facility location problem. Due to its intrinsic problem complexity, researchers have turned to various heuristics to guide the selection. Different applications may impose diverse service requirements; hence, the service selection should follow distinctive criteria to achieve the best service performance. In [24], Andersen et al. demonstrated that relay nodes are able to improve network resilience; however, the manual and static relay node selection is not scalable. Random selection is light-weighted; however, it has only achieved limited success [25]. In [26], a heuristic randomization relay selection algorithm with the assistance of end-to-end path probing was proposed for improving end-to-end average round-trip delay. In [11], Liu et al. proposed to break an end-to-end path with a large RTT into multiple sequential TCP connections with smaller local RTTs for increasing the end-to-end TCP throughput. Similar studies on improving TCP throughput using relays can be found in [27–29]. The joint optimization problem of multiple relay selection and the rate control problem was studied in [30]. Chen et al. proposed a heuristic one-hop relay node selection (HORNS) for supporting the interaction of the real-time applications in [31]. Hei and Song proposed a stochastic routing framework for selecting relay nodes in P2P networks [32]. In [33], Zhang et al. further extended the stochastic routing problem and proposed a dynamic stochastic routing algorithm in overlay networks for better accuracy. In [34], Zhang et al. considered the implementation issues how to approximate the overlay link delay distribution via measurements. In [35], Cohen and Raz studied the overlay routing resource allocation (ORRA) problem. They proved that it is a NP hard problem and proposed a non-trivial approximation algorithm to solve the sub-optimization problem: find a minimal set of overlay nodes such that the required routing properties are satisfied. The results show that a relative small number of less than 100 relay servers are sufficient to enable shorter paths in the two practical scenarios, namely BGP routing and TCP improvement. These research work focused on finding the relay nodes to build the intermediate paths without modifying the overlay topology structure.

Previous researchers have also considered various effective methods to handle the relay paths by placing the relay nodes directly in overlay networks [36–39]. These research examined more metrics in choosing the better relay nodes among the relay backup subsets, such as the routing traffic [36], link bandwidth [37], TCP performance [38] or link available bandwidth [39], to build the optimal relay paths.

Network locations of the relays are crucial parameters to conduct a successful relay selection. Nevertheless, in these studies, those selection parameters are either assumed to be given in advance or they are measured via direct probing. In a large-scale P2P network, thousands of relays may serve as relay candidates. Extensive direct probing to a large number of relays is prohibited because the measurements lead to significant traffic overhead and the measurement results may not be readily available before the selection. An interesting recent work reports that compared to heavy fine-grained measurements, coarse-grained path ratings with small overhead are able to provide locality-aware overlay construction and routing with good accuracy [40]. Those path ratings can be acquired by statistical inference based on matrix factorization techniques. To address the difficulty of direct probing, indirect measurement on relays’ network location has been explored for assisting the selection.

Utilizing the DNS server or the network topology information, such as the Autonomous System (AS) topology, have been popular to estimate network location. For example, the King tool [18] estimates the delay between two DNS servers and approximates it as end-to-end delays between two end-hosts. The King measurements are error-prone when the end-hosts are far away from their DNS servers or the network condition changes. The AS information has also been explored for assisting the relay selection in [16]. In [16], Fei et al. proposed to select the relay nodes to identify an alternative path that are as disjoint as possible from the default path at the AS level. Both [17] and [41] focused on utilize the efficient relay node selection to improve the performance of the P2P-based VoIP applications. In [17], Ren et al. proposed an AS-Aware Peer-Relay(ASAP) protocol to shorten the RTT between the caller and the callee. [41] proposed another relay selection algorithm for improving ASAP. There have been proposed a few Internet Coordinate Systems (ICS) including GNP [13], Landmarks [14], and Vivaldi [15]. The limitations on the measurement accuracy of the existing ICSes was examined in [42] and the ICS accuracy may be improved using subspace embedding. A comprehensive survey of ICS can be found in [43]. In our study, we evaluate the selection accuracy of relays based on the indirect RTT measurement using King and Vivaldi.

## 8 Conclusion

In this paper, we study the location-aware relay discovery and selection problem for large-scale streaming. Indirect measurements, i.e., King and ICS, enjoy small measurement overhead but suffer from significant errors. The impact of the measurement errors will be further amplified in the general “best-out-of-*K*” selection. Nevertheless, the indirect measurements can still be used to narrow down a small number of relay candidates efficiently. Based on an extensive measurement study, we show that a two-phase approach with combined indirect measurement and direct probing is able to achieve efficient relay discovery and accurate relay selection. This two-phase approach can be implemented efficiently using the DHT.

This paper focuses only on the one-hop relay selection for large-scale P2P streaming networks from the practical perspective. It would be interesting to further study *N*-hop relay (*N* > 1) cases. This study is essentially an empirical study which heavily relies on experimental data with limited theoretical proof and analysis; hence, another directions worth further research include conducting the theoretical error analysis to better understand the performance bound of the proposed practical relay discovery and selection scheme.
